# 

**Published:** 1860-11

**Authors:** 


### CONTENTS FOR NOVEMBER, 1860.

### ORIGINAL COMMUNICATIONS.

Report on the Medical Uses of Veratrum Viride. By A. HARD, M.D., of Aurora.... 641
Report of the Committee on Practical Medicine. By C. GOODBRAKE, M.D., of Clinton 649

### BOOK AND PAMPHLET NOTICES.

Remarks on a Review of Dr. BRINTON'S Work, entitled " Ulcer of the Stomach," by the " American Journal of Medical Sciences," for July, 1859. By T. DEVILLE, M.D., Associate of King's College, London, late of Ecole Partique, Paris, and Professor of Anatomy, Lind University ........ 688

### CLINICAL REPORTS.

Clinical Cases. By THE EDITOR........ 689—690

### SELECTIONS.

New York Academy of Medicine. Discussion on Tetanus........ 691
" The American Medical Monthly and New York Reviewer." Clinical Researches on the Action of Diuretic Remedies, by AUSTIN FLINT, M.D........ 695
On the Prevention of Pitting in Confluent Small-Pox. By WILLIAM STOKES, M D., Regius Professor of Physic in the University of Dublin........ 696

### EDITORIAL.

Medical Schools in Chicago........ 698—699
Chicago Academy of Medical Sciences. Regular Meeting, Nov. 2nd, 1860........ 700
Scott County Medical Society........ 701

